# Isolation, Genomic Characterization, and Pathogenicity of a Novel Duck Orbivirus Genetically Similar to Corriparta Virus in China

**DOI:** 10.1155/tbed/9556666

**Published:** 2025-11-24

**Authors:** Liping Liu, Xiaozhen Guo, Feng Hu, Xiao Lu, Yumiao Lv, Jianhua Wang, Qin Ma, Yigang Tong, Fengjuan Tian, Yingjuan Qian, Yong-Sam Jung, Xiuli Ma, Bing Huang, Yufeng Li, Kexiang Yu

**Affiliations:** ^1^Institute of Poultry Science, Shandong Academy of Agricultural Sciences, Jinan 250100, Shandong Province, China; ^2^Shandong Provincial Key Laboratory of Livestock and Poultry Breeding, Institute of Poultry Science, Shandong Academy of Agricultural Sciences, Jinan 250100, Shandong Province, China; ^3^Research and Development Department, Shandong Hekangyuan Biological Breeding Co., Ltd., Jinan 250023, Shandong Province, China; ^4^State Key Laboratory of Green Biomanufacturing, College of Life Science and Technology, Beijing University of Chemical Technology, Beijing 100029, China; ^5^Laboratory of Emerging Infectious Diseases and One Health, College of Veterinary Medicine, Nanjing Agricultural University, Nanjing 210095, Jiangsu Province, China

**Keywords:** Corriparta virus, duck, genomic characterization, pathogenicity

## Abstract

Corriparta virus (CORV), an arbovirus within the *Orbivirus* genus, exhibits a broad vertebrate host range but limited pathogenicity. In this study, we report the first isolation and characterization of a novel orbivirus genetically related to CORV, temporarily designed as novel duck orbivirus (NDORV), from Beijing ducks in Henan province, China, in 2024. Genomic characterization revealed that NDORV possesses a 10-segment double-stranded RNA (dsRNA) genome, consistent with the structural hallmarks of the *Orbivirus* genus, with a high genetic similarity to Parry's Lagoon virus (PLV) and CORV. To evaluate its pathogenicity, specific pathogen-free (SPF) ducks were experimentally inoculated with NDORV. Gross pathological examination revealed splenomegaly and blood stasis as primary lesions, with no mortality observed. Histopathological analysis identified tissue damage in the spleen, lungs, heart, liver, and kidneys. The highest viral loads were observed in the spleen and lungs, peaking at 3 days postinoculation (dpi). This study provides the first comprehensive characterization of a novel orbivirus genetically akin to CORV isolated from ducks in China. These findings highlight the potential prevalence of NDORV in domestic duck populations and underscore the urgency of enhanced surveillance and research on CORV-related arboviruses.

## 1. Introduction

Viruses of the *Reoviridae* family typically possess a 9–12 segmented double-stranded RNA (dsRNA) genome. These viruses are nonenveloped and exhibit an icosahedral symmetry, with a diameter ranging from 60–80 nm. They are widely distributed in nature and infect a broad range of hosts, including vertebrates, invertebrates, and plants. The *Reoviridae* family is classified into 15 distinct genera, with the genus *Orbivirus* being the largest [1–4]. Orbiviruses include 22 distinct virus species. Among which, bluetongue virus (BTV), African horse sickness virus (AHSV), epizootic hemorrhagic disease virus (EHDV), and equine encephalosis virus (EEV) are considered the most economically significant. These viruses cause substantial economic losses due to herd morbidity and mortality, as well as restrictions on animal movement and trade [5–9].

Orbiviruses are characterized by a 10-segment dsRNA genome, two shell layers and an icosahedral structure. Within the *Orbivirus* genus, the most conserved proteins are VP1 (Pol), T2, and T13, which are commonly used for genus-level identification. In contrast, OC1 (VP2/3) and OC2 (VP5) are highly variable and are often utilized for serotype differentiation within the genus [10–12]. Significantly, the high conservation of VP1 makes it a reliable target for sequencing and RT–PCR assays, enabling the identification of virus isolates at both the species and genus levels [1, 13].

Corriparta virus (CORV), a member of the *Orbivirus* genus, is an arthropod-borne virus (arbovirus) primarily transmitted by mosquitoes. It has a wide geographic distribution and host range, being primarily prevalent in Australia, Africa, and South America, with additional reports in the United States [14, 15]. Although no overt disease has been observed in infected hosts, CORV-neutralizing antibodies have been detected in a wide range of vertebrate species, including wild and domestic birds, cattle, marsupials, horses, and humans [16–19]. This broad seroprevalence highlights the extensive host range of CORV and its potential for cross-species transmission, despite the absence of clinical symptoms in most cases. CORV is sensitive to low pH and heat, and its infectivity can be modified by treatment with trypsin or chymotrypsin [20].

CORV was first isolated from *Culex annulirostris* mosquitoes collected in North Queensland, Australia, in 1960. Currently, the International Committee on Taxonomy of Viruses (ICTV) recognizes six serotypes/strains of CORV: CORV-MRM1; CS0109; V654; V370; Acado virus and Jacareacanga virus [14, 18, 21]. In 2013, an additional member, California Mosquito Pool virus (CMPV), isolated in 1974, was assigned to the CORV species [22]. Due to the lack of representative sequences from all CORV strains for comparison, it was initially considered to be a new species within the *Orbivirus* genus. In 2016, Harrison et al. [23] reported the discovery of Parry's Lagoon virus (PLV) in *Culex annulirostris*, *Culex pullus*, Mansonia uniformis, and *Aedes normanensis* in northern Australia. Neutralizing antibodies against PLV have been detected in various mammalian species, but the virus could only be isolated in C6/36 cells and failed to replicate in cells of vertebrate origin, despite its close genetic and antigenic relatedness to CORV [23].

Although CORV has a relatively limited impact on public health compared to other arboviruses, it remains an important subject of study due to its potential to cause disease and its ecological significance in mosquito–vertebrate transmission cycles. In this study, we report, for the first time, the isolation of a novel virus belonging to the *Orbivirus* genus, tentatively named novel duck orbivirus (NDORV), from Beijing ducks in Henan province, China, in 2024. Genomic analysis revealed that this virus shares significant homology with CORV. Animal experiments confirmed that NDORV exhibits moderate pathogenicity in specific pathogen-free (SPF) ducks. This study serves as an early warning that the virus may be circulating in duck populations and could pose a potential threat.

## 2. Materials and Methods

### 2.1. Clinical Sample Collection and Treatment

In 2024, the breeding ducks in a farm in Henan Province of China had symptoms with decreased egg production and poor eggshell quality. Postmortem examination exhibited signs of splenomegaly or even rupture, salpingitis, and peritonitis. Samples from the trachea, spleens, and fallopian tubes of the diseased ducks were collected and homogenized. The negative results of the common duck pathogens (e.g., duck hepatitis virus, duck enteritis virus, avian influenza virus, Newcastle disease virus, duck tembusu virus, duck reovirus, duck parvovirus, duck astrovirus, and duck circovirus) indicated that a novel or unidentified agent might be existed.

### 2.2. Virus Isolation and Propagation

The samples were filtered through a 0.22 µm membrane and then inoculated into the yolk sac of 7-day-old SPF duck embryos. The inoculated embryos were incubated at 37°C for 7 days. After the incubation period, the allantoic fluids and infected embryos were harvested, homogenized, and centrifuged to clarify the supernatant, which was then employed for subsequent propagation in SPF duck embryos. Blind passage was conducted for five generations.

The duck embryo fibroblast (DEF) cells were cultured in Dulbecco's Modified Eagle Medium (DMEM, Gibco, USA) supplemented with 10% fetal bovine serum (FBS, PAN, Germany) and used for further virus isolation and characterization. After virus adsorption at 37°C for 1 h, cells were maintained in DMEM containing 1% FBS. When 80% cytopathic effect (CPE) was observed, the infected cell cultures underwent two freeze–thaw cycles to release viral particles. The supernatant was subsequently harvested and stored at −80°C. This procedure was repeated for five sequential passages to amplify the viral titer.

### 2.3. Viral Replication Kinetics in DEF Cells

DEF cells seeded in 24-well plates at 90% confluence were inoculated with NDORV at an MOI of 0.1. After 1 h of adsorption per in a 37°C CO_2_ incubator, the cells were washed three times with PBS, followed by the addition of 0.5 mL maintenance medium per well. At 2, 12, 24, 48, 72, 96, and 120 h postinoculation (hpi), both cells and supernatants were collected, and NDORV titers were determined using the 50% tissue culture infective dose (TCID_50_) method.

### 2.4. Transmission Electron Microscopy (TEM) Observation

DEF cells were inoculated with NDORV and gently scraped from the culture flask on the 2-day postinoculation (dpi). The cell cultures were harvested and centrifuged at 8000 × *g* for 20 min at 4°C, and the supernatant was discarded. The cell pellet was fixed in 3% glutaraldehyde. The fixed cells were processed using conventional TEM sample preparation methods, including rinsing, secondary fixation in 1% osmium tetroxide (OsO_4_), further rinsing, dehydration, infiltration, and embedding in Epon812. Ultrathin sections (70–100 nm) were cut using an LKB-V ultramicrotome. These sections were stained with lead citrate and uranyl acetate and examined under a JEOL-1200E TEM, with images captured using a MORADA-G2 camera.

### 2.5. Genome Sequencing and Phylogenetic Analysis

The cells infected with NDORV were collected and subjected to one freeze–thaw cycle, followed by centrifugation at 10,000 × *g* for 20 min. The clarified supernatant was sent to a commercial service provider (Sangon Biotech, Shanghai, China) for next-generation sequencing (NGS) analysis to determine the complete genomic sequence of the virus.

Sequences of representative orbiviruses were retrieved from NCBI to conduct phylogenetic analysis. Based on the amino acid sequences of viral proteins, phylogenetic trees for each protein were constructed using MEGA 7 software via the neighbor-joining method. To ensure the reliability of the tree topology, a bootstrap analysis with 500 replications was performed. Subsequently, the phylogenetic trees were further refined and visualized as heatmaps using an online tool (https://www.chiplot.online/tvbot.html).

Following the assembly of the genome, a comprehensive comparison was carried out between the assembled genome and the reference sequences of PLV (K71551 strain) and CORV (AUS1960/01 strain). The comparison included the name of the encoded protein, G + C content (%), the length of the open reading frame (ORF), predicted protein length (aa), nucleotide homology, and amino acid homology.

### 2.6. Quantitative Reverse Transcription PCR (qRT-PCR) Detection

The qRT-PCR assay was performed according to the Multiplex One-Step qRT-PCR Probe Kit instructions (GenStar, CHN), which contains 100 nM of each primer NDORV-F/R and NDORV-probe (Table 1), 2 μL of RNA template. Plasmid standards were constructed to establish a standard curve for viral load quantification. The reaction conditions were reverse transcription at 50°C for 10 min, initial denaturation at 95°C for 2 min, 40 cycles at 95°C for 10 s, and final extension at 60°C for 30 s. The sensitivity was up to 100 copies/μL.

### 2.7. Pathogenicity Tests

#### 2.7.1. Grouping of Experimental Animals

Around 2-day-old SPF ducks were randomly divided into two groups, with 30 ducks in each group, and housed in separate isolators under similar conditions. The inoculation group received an intramuscular injection of 0.1 mL suspension (TCID_50_ = 10^5^/0.1 mL), while the control group received an equivalent amount of normal duck embryo homogenate. Clinical changes in the SPF ducks were monitored and recorded daily for 28 days. Body weights of seven designated ducks from each group were measured at 0, 3, 7, 14, 21, and 28 dpi. Cloacal and oral swabs were collected daily from 1 to 7 dpi and stored at −80°C for subsequent analysis. Furthermore, blood samples were obtained at 10, 20, and 30 dpi. The serum samples separated from these collections were stored at −20°C for future use.

#### 2.7.2. Anatomical Observation and Sectioning

In both the inoculation and control groups, three SPF ducks were randomly selected and euthanized for dissection to examine organ lesions on 3, 7, 14, 21, and 28 dpi. The heart, liver, spleen, lungs, kidneys, and bursa of Fabricius were collected and weighed to calculate the organ coefficients (organ weight (g)/body weight (g) × 100%). A portion of each organ tissue was fixed for histopathological sectioning. The remaining tissues from the three ducks were pooled in equal weights, homogenized, and stored at −80°C for further analysis.

#### 2.7.3. Viral Load in Different Tissues, Oral Swabs, and Cloacal Swabs

Total RNA was extracted from the organs, oral swabs, and cloacal swabs of experimental ducks using the MiniBEST Viral RNA/DNA Extraction Kit (Beijing Takara Biotech Co., Ltd.) following the manufacture's instructions. A previously established qRT-PCR assay was employed to quantify the viral load in each sample. The copy number of the virus was calculated by substituting the Ct values from the inoculation group into a standard curve. Each sample was analyzed in triplicate, and the mean value was determined. The experimental data were plotted to visualize the viral distribution upon different organs at different time points.

#### 2.7.4. Neutralization Assays

Three serum samples from each time point were heat-inactivated at 56°C for 30 min to eliminate nonspecific antiviral activity. The inactivated sera were subjected to two-fold serial dilutions and mixed with an equal volume of NDORV containing 200 TCID_50_. The virus-serum mixtures were incubated at 37°C for 1 h to allow neutralization. Subsequently, 0.1 mL of each mixture was inoculated onto DEF cell monolayers in 96-well plates, with six replicate wells per mixture to ensure statistical reliability. The plates were incubated at 37°C with 5% CO_2_ for 120 h. Cells were examined daily for the presence of CPE. The neutralizing antibody titer for each sample was defined as the reciprocal of the highest serum dilution that protected more than 50% of the cells from CPE, as calculated by the Reed–Muench method.

### 2.8. Statistical Analysis

Results are expressed as the mean ± standard deviation. Three analyses were conducted using Student's *t*-test with GraphPad Prism 6 software (GraphPad Software, San Diego, CA, USA). A *p*-value of less than 0.05 was considered statistically significant for group differences.

## 3. Results

### 3.1. Virus Isolation and Confirmation

**Figure 1:**
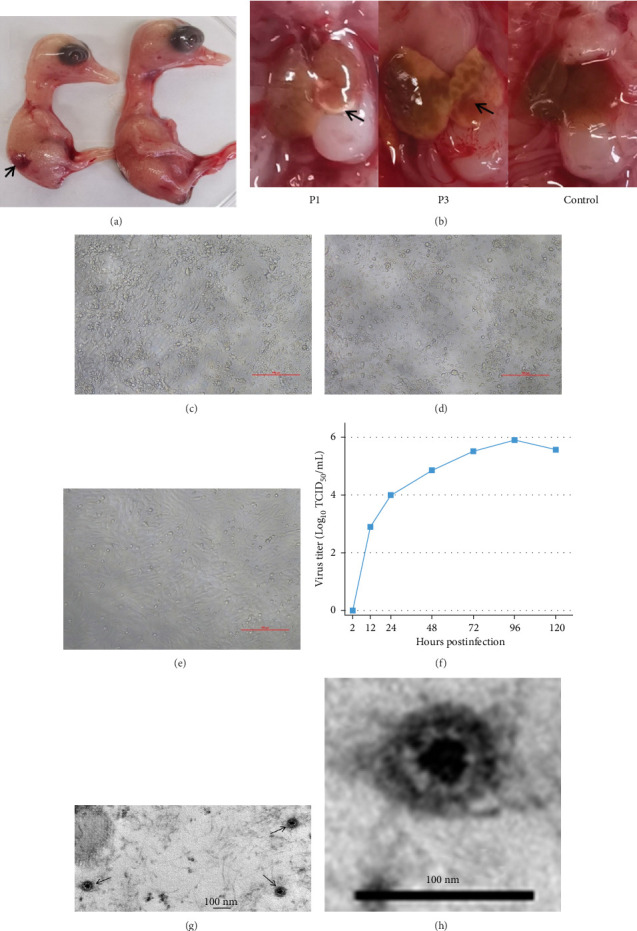
Virus isolation and confirmation. (A) Pathological changes in SPF duck embryos. The left embryo was inoculated with NDORV, while the right embryo injected with PBS serves as the control. (B) Liver lesions in embryos infected with NDORV at the first (P1) and third (P3) passages. (C) Initial CPE in DEF cells at Passage 1. (D) Extensive CPE in DEF cells at Passage 5. (E) Control DEF monolayer treated with PBS. (F) Viral replication kinetics of NDORV in DEF cells, as determined by TCID_50_. (G,H) TEM images of NDORV particles. Scale bars: 100 nm.

Following five blind passages in SPF duck embryos, NDORV induced only limited mortality in the embryos. However, necrotic lesions were observed at the edges of the embryonic livers starting from the first passage. By the third passage, the infected SPF duck embryos exhibited additional pathological changes, including smaller embryo size, hemorrhages, and extensive liver necrosis (Figure 1A,B).

The propagation of the virus in DEF cells induced CPE as early as the first passage, characterized by cellular rounding, shrinkage, and fragmentation, with ~30% of the cell area affected (Figure 1C). With subsequent serial passaging, the CPE intensified in both severity and spatial distribution, ultimately affecting about 80% of the monolayer by the fifth passage (Figure 1D). In contrast, mock-infected control cells maintained a normal morphology throughout the experiment (Figure 1E). These findings confirm the successful isolation and amplification of the virus in DEF cells, establishing a platform for characterization of the viral replication kinetics and pathogenic mechanisms.

The replication kinetics of NDORV in DEF cells are shown in Figure 1F. The infectious virus was first detected at 12 hpi, after which the titer increased in a time-dependent manner, peaking at 10^5.9^ TCID_50_/mL by 96 hpi. Thereafter, the titer began to decline by 120 hpi.

Small, icosahedral and non-enveloped particles were observed under TEM (Figure 1G,H). The virus particles are ~60 nm in diameter, aligning with the typical size range for *Reoviridae* family viruses. The TEM images confirmed particles exhibiting characteristic *Reoviridae* morphology.

### 3.2. Genome Sequence, Organization, and Phylogenetics

The sequencing data were assembled with the PLV genome serving as the reference sequence. The complete genome sequence (Accession number: PV797989-PV797998) was obtained, which consists of 10 segments. Each ORF within these segments was comprehensively characterized. It was found that 8 out of the 10 segments (seg-1 to seg-8) contain a single ORF, while the remaining two segments (seg-9 and seg-10) have two overlapping ORFs. These overlapping ORFs encode the proteins VP6/NS4 and NS3/NS3a, respectively, which is in line with the typical genomic structure of orbiviruses.

**Figure 2:**
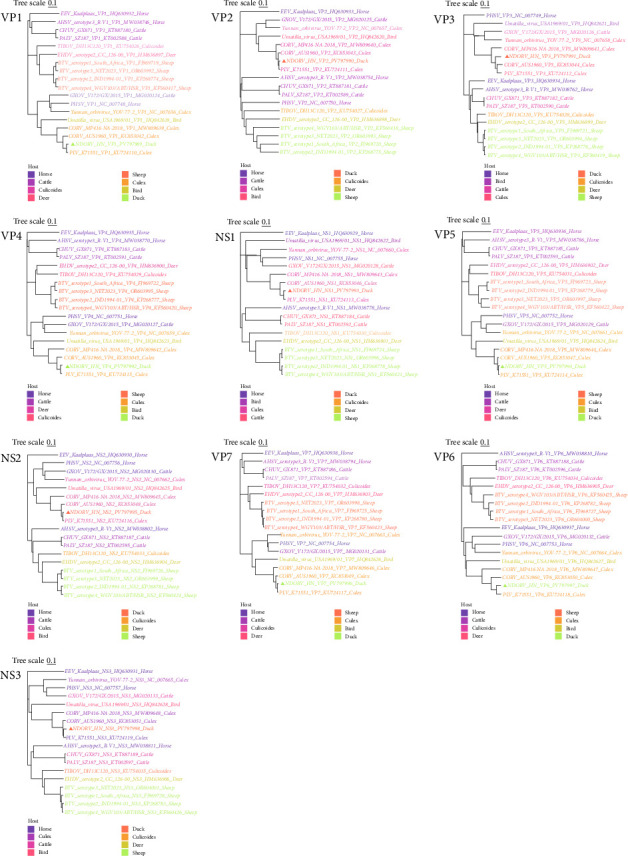
Phylogenetic analysis of NDORV. The phylogenetic tree based on each protein encoded by the NDORV genome was constructed using the neighbor-joining method in MEGA 11 with bootstrap values from 500 replicates and was refined using https://www.chiplot.online/tvbot.html. Lineages with different hosts are indicated by color blocks, and NDORV is marked with a black box.

A phylogenetic tree was constructed to analyze the evolutionary relationships. The results indicated that each protein encoded by the NDORV genome clusters on the same small branch as those of PLV and CORV, both of which were isolated from Culex mosquitoes (Figure 2).

Pairwise alignments were performed between the ORFs of the novel isolate and those of PLV and CORV, and the results are presented in Table 2. The 12 proteins of the novel virus exhibit high similarity to the K71551 strain of PLV, with amino acid homologies ranging from 82.4% to 98.4%, and to the AUS1960/01 strain of CORV, with amino acid homologies ranging from 74.7% to 95.4%.

### 3.3. Pathogenicity and Clinical Manifestations of NDORV in SPF Ducks

**Figure 3:**
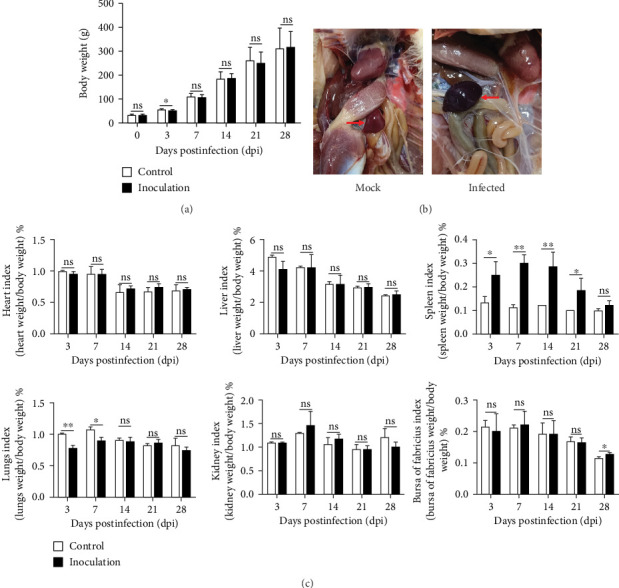
Pathogenicity and clinical manifestations of NDORV in SPF ducks. (A) Body weight changes in SPF ducks following NDORV infection. (B) Observation of changes in spleen after dissection. (C) Organ coefficients in SPF ducks following NDORV infection. Data are presented as mean ± standard deviation (SD). *⁣*^*∗*^*p* < 0.05, *⁣*^*∗∗*^*p* < 0.01, ns means *p* ≥ 0.05.

Throughout the study, all SPF ducks remained in good general condition, with normal mental status, feed intake, and water consumption. No clinical signs—including diarrhea, respiratory distress, lethargy, or anorexia—were observed at any time postinoculation. Notably, all birds survived, resulting in a 100% survival rate. Seven ducks from each group were weighed at 0, 3, 7, 14, 21, and 28 dpi. The data demonstrated that the body weights of the inoculated group were comparable to those of the control group at all time points, except for a slight deviation observed at 3 dpi (Figure 3A). This indicates that the virus had no significant impact on the growth or overall health of the ducks.

Three SPF ducks from each group were randomly selected and euthanized at 3, 7, 14, 21, and 28 dpi. The autopsy revealed that only the spleen has visual lesions such as enlargement and blood stasis (Figure 3B). The organ coefficients were calculated and compared between the inoculated and control groups. The results showed that the spleen coefficient of the inoculated group increased significantly at 3, 7, 14, and 21 dpi, and the difference was not significant at 28 dpi. The lung coefficients of the inoculated group showed a marked decrease at 3 and 7 dpi, after which the difference was not significant. And no significant differences were observed in the other detected organs (Figure 3C).

**Figure 4:**
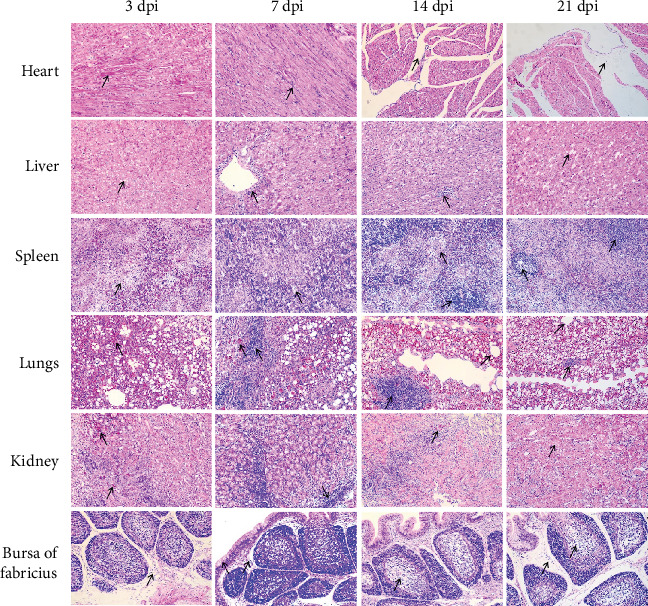
Histopathologic changes in organs of SPF ducks infected with NDORV. Histopathologic changes in organs were examined at 3, 7, 14, and 21 dpi. All images were obtained using hematoxylin and eosin (H&E) staining at 200x magnification.

Histopathologic changes indicated the NDORV predominantly affecting the heart, liver, spleen, lungs, and kidneys of SPF ducks. The myocardial cells exhibited marked granular depassage, and the interstitial spaces between myocardial fibers were significantly widened. Additionally, there was a noticeable increase in fibroblasts within the myocardial interstitium, accompanied by mild inflammatory cell infiltration. The hepatic architecture was disrupted, with loss of clarity in the hepatic lobule and cord structures. Under high magnification, hepatocyte swelling and focal hepatocyte necrosis were observed, accompanied by mild inflammatory cell infiltration. The spleen exhibited significant reduction of white pulp, accompanied by a marked decrease in lymphocytes within the white pulp and an increase in interstitial components. Focal necrosis was observed in some regions. Additionally, there was focal infiltration of inflammatory cells, with prominent lymphoid follicles in certain areas and thickening of the periarteriolar lymphoid sheaths. The lung tissue exhibited congestion, hemorrhages, and focal infiltration of inflammatory cells. Additionally, overexpansion of some alveoli was observed. The kidney tissue exhibited focal hemorrhages, along with extensive infiltration of heterophils and focal infiltration of lymphocytes in localized areas. These histopathologic changes provide visual evidence of the tissue damage caused by NDORV infection (Figure 4).

### 3.4. Systemic Viral Dissemination and Humoral Immune Response to NDORV in SPF Ducks

**Figure 5:**
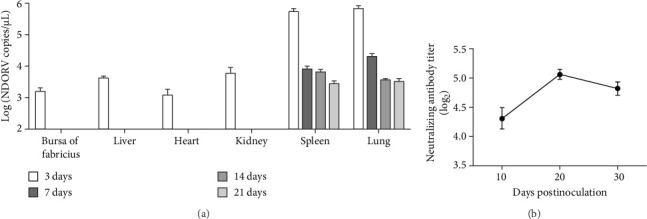
Viral load in different tissues and serum neutralizing antibody titers of SPF ducks infected with NDORV. (A) The viral load in different tissue organs was examined using a qRT-PCR method at 3, 7, 14, 21, and 28 dpi. (B) Serum neutralizing antibodies titers were determined at 10, 20, and 30 dpi Data are presented as mean ± SD.

The viral loads in different tissues, oral swabs, and cloacal swabs were examined using an established qRT-PCR method. The results showed that the highest viral loads were found in the spleen and lung, which reached their peak at 3 dpi with 5.49 × 10^5^ copies/μL and 6.39 × 10^5^ copies/μL, respectively. The viral load gradually decreased to 2.67 × 10^3^ copies/μL in the spleen and 3.21 × 10^3^ copies/μL in the lung at 21 dpi. No viral RNA was detected in these two organs at 28 dpi. Additionally, viral loads were detected in the heart, liver, kidney, and bursa Fabricius only at 3 dpi, indicating transient viral replication in these tissues (Figure 5A). Viral shedding was observed exclusively via the cloacal route, beginning at 2 dpi with all seven ducks (7/7) testing positive. This declined to 2/7 positive ducks by 3 dpi and ceased entirely by 4 dpi (Table 3). No viral RNA was detected in oral swabs throughout the study. These results illustrated the temporal changes in viral replication and clearance.

Serum neutralizing antibodies titers were evaluated at 10, 20, and 30 dpi. The geometric mean titer increased from 2^4.32^ at 10 dpi to a peak of 2^5.08^ at 20 dpi, and subsequently declined at a slightly lower level of 2^4.83^ by 30 dpi (Figure 5B), indicating the induction of a humoral immune response following infection.

## 4. Discussion

The genus *Orbivirus*, belonging to the subfamily *Sedoreovirinae* within the family *Reoviridae*, comprises vector-borne pathogens transmitted by ticks, mosquitoes, gnats, and midges. Among the orbiviruses, several members are known to infect animals and cause significant diseases, including BTV, AHSV, EHDV, and EEV [5]. BTV causes bluetongue disease in ruminants such as cattle, sheep, and goats, leading to severe economic losses in livestock industries [24–26]. AHSV affects horses, cattle, and sheep, leading to African horse sickness, a highly fatal disease [27]. EHDV infects deer, cattle, and sheep, causing epizootic hemorrhagic disease, which can cause high mortality in wild and domestic ruminants [7]. EEV is associated with equine encephalosis in horses, a neurological disease affecting horses [28, 29]. Orbiviruses have a wide range of hosts, including birds [23], but there have been no reports of orbiviruses causing disease in poultry. In contrast, infections by viruses of the genus *Orthoreovirus*, which belong to the same family (*Reoviridae*), are common in poultry and can lead to significant diseases. For example, avian reovirus causing viral arthritis in chickens, muscovy duck reovirus leading to hepatic white spot disease in Muscovy ducks and geese, and novel duck reovirus associating with spleen necrosis disease in ducks [30–32]. However, these viruses are genetically distant from NDORV. As a member of the *Orbivirus* genus, CORV can infect a variety of animals, including humans, but it has not been associated with any pathogenic effects to date. Notably, studies have reported that CORV can replicate efficiently in Muscovy duck cells (AGE1.CR) [33], but there have been no reports of its isolation from ducks.

In this study, we report the first isolation of an orbivirus from ducks, which causes hemorrhages and liver necrosis in duck embryos. The full genome of the virus was sequenced, analyzed, and compared with other orbiviruses. The results revealed that the genome of this virus shares the highest homology with PLV (K71551) available in GenBank, with nucleotide sequence identity ranging from 72.8% to 92.8% and amino acid sequence identity ranging from 82.4% to 98.4%. PLV, a novel circovirus isolated from Culex mosquitoes in Australia, is genetically and antigenically similar to CORV and is considered a CORV but cannot replicate in several vertebrate cell lines suitable for CORV propagation, such as DF-1, BHK, and Vero cells [23]. Additionally, the genome of NDORV shows relatively high homology with CORV (AUS1960/01), with nucleotide sequence identity ranging from 72.0% to 82.0% and amino acid sequence identity ranging from 74.7% to 95.4%.

Currently, we have detected NDORV in multiple populations of duck species with clinical symptoms, most of which exhibited pathological changes, such as spleen enlargement or even rupture. These findings are consistent with the symptoms observed in SPF ducks experimentally infected with NDORV, which also developed spleen enlargement. Notably, reduced egg production was observed in some NDORV-positive duck flocks, and qRT-PCR analysis identified the oviduct as the site with the highest viral load. However, the direct impact of NDORV on reproduction remains unclear, as this study did not include systematic reproductive parameters or histopathological examination of ovarian and oviduct tissues. Whether NDORV has the potential to affect avian reproductive performance requires further investigation through well-controlled challenge experiments, longitudinal egg production monitoring, and detailed histopathological analysis. As a newly identified virus, multiple aspects of NDORV, including its origin, transmission routes, pathogenicity, and mechanisms of genetic variation, require further investigation. Additionally, its potential to infect other animal species or even humans remains unclear and warrants additional exploration.

## 5. Conclusion

We present the initial isolation and identification of a NDORV from diseased ducks with distinct clinical manifestations. Genomic analysis revealed that this virus has a remarkable degree of homology with CORV. Animal experiments confirmed that NDORV demonstrates moderate pathogenicity in SPF ducks, with the primary symptom being spleen enlargement. This study serves as an early alert, suggesting that the virus might be circulating within duck populations and potentially endangering duck health.

## Figures and Tables

**Table 1:** Primer and probe sequences of NDORV.

Name	Sequences (5′-3′)	Length of target gene
NDORV-F	GCTTGGTATGATGCTCTAC	143 bp
NDORV-R	GGTGATACTAAGATTGGTGTAC	
NDORV-probe	FAM-CGTAGTTCCTGAGGATGCACCGTTC-TAMRA	

**Table 2:** NDORV genome characteristics.

Segment	Protein	G + C (%)	ORF (bp)	Predicted protein length (aa)	% Pairwise identity with PLV (K71551) (nt/aa)	% Pairwise identity with CORV AUS1960/01 (nt/aa)
1	VP1	41.4	3873	1290	89.7/97.4	77.4/91.2
2	VP2	42.1	2850	949	90.9/98.4	80.4/95.4
3	VP3	41.9	2220	739	72.8/82.4	72.0/80.2
4	VP4	43.7	1932	643	88.1/96.1	78.7/90.8
5	NS1	48.3	1770	589	91.5/96.6	78.0/89.5
6	VP5	45.5	1584	527	88.8/97.9	79.3/95.3
7	NS2	48.0	1116	371	92.8/98.1	81.0/92.7
8	VP7	49.8	1065	354	88.2/98.3	79.2/95.2
9	VP6	47.1	1035	344	85.3/84.0	80.2/74.7
	NS4	47.7	459	152	84.7/86.8	79.3/79.6
10	NS3	46.7	717	238	89.3/94.1	82.0/93.3
	NS3a	48.0	327	108	89.9/91.7	81.3/90.7

**Table 3:** Viral shedding detection in NDORV-challenged ducks.

Days postinoculation	Number of positive ducks (positive/total)	
	Cloacal swab	Oral swab
1	0/7	0/7
2	7/7	0/7
3	2/7	0/7
4	0/7	0/7
5	0/7	0/7
6	0/7	0/7
7	0/7	0/7

## Data Availability

The data used to support the findings of this study are available within the paper.
